# Regional variation in antibiotic prescribing among medicare part D enrollees, 2013

**DOI:** 10.1186/s12879-016-2091-0

**Published:** 2016-12-09

**Authors:** Andre Arizpe, Kelly R. Reveles, Samuel L. Aitken

**Affiliations:** 1 College of Pharmacy, The University of Texas at Austin, Austin, TX USA; 2 Pharmacotherapy Education and Research Center, The University of Texas Health Science Center at San Antonio, San Antonio, TX USA; 3 Division of Pharmacy, The University of Texas MD Anderson Cancer Center, 1515 Holcombe Blvd, Unit 0090, Houston, TX 77030 USA; 4 Center for Antimicrobial Resistance and Microbial Genomics, UTHealth McGovern Medical School, Houston, TX USA

**Keywords:** Antibiotic stewardship, Fluoroquinolones, Elderly, Macrolides, Claims data, Outpatient prescriptions, Cephalosporins, Penicillins

## Abstract

**Background:**

Antibiotics are among the most widely prescribed medications. The geographic variation in antibiotic prescribing patterns and associated costs among Medicare Part D recipients have not been described. The purpose of this study was to assess the regional variation in antibiotic prescriptions and costs among Medicare Part D enrollees in 2013.

**Methods:**

Retrospective cohort review of all Medicare Part D enrollees in 2013, using the Medicare Provider Utilization and Payment Data: Part D Prescriber Public Use File. All original or refill prescription claims for antibiotics as listed in the Part D Prescriber Public Use File were included. Our primary outcomes were total antibiotic claims and antibiotic cost per Medicare Part D Enrollee. Data were analyzed descriptively by state and by geographic region as defined by the United States Census Bureau. Antibiotic claims were described overall and by antibiotic class.

**Results:**

Over 54 million outpatient antibiotic claims were filed for Part D enrollees in 2013, representing more than $1.5 billion in total antibiotic expenditures. Antibiotic use was highest in the South (1,623 claims/1,000 enrollees), followed by the Midwest (1,401 claims/1,000 enrollees), Northeast (1,366 claims/1,000 enrollees), and West (1,292 claims/1,000 enrollees). Average antibiotic costs per enrollee in each region were as follows: South $46.58, Northeast $43.70, Midwest $40.54, and West $36.42. Fluoroquinolones were the most commonly prescribed class overall (12.2 million claims).

**Conclusions:**

Antibiotic use among elderly Medicare enrollees in the United States was highest in the South region. Fluoroquinolones were the most common antibiotics used in all regions. These patterns could be utilized in the development of targeted antimicrobial stewardship efforts.

**Electronic supplementary material:**

The online version of this article (doi:10.1186/s12879-016-2091-0) contains supplementary material, which is available to authorized users.

## Background

The advent of clinically useful antibiotics revolutionized the practice of medicine; however, the emergence of antibiotic-resistant organisms has posed a unique challenge to the utilization of these drugs. Despite advances in drug development and infection control measures, antibiotic resistance continues to be a great concern [1]. Each year in the United States, antibiotic-resistant infections are responsible for an estimated 23,000 deaths and as much as $20 billion in direct healthcare costs [2].

The overuse of antibiotics is a major factor in the development of resistant organisms [3]. It is estimated that 30% of outpatient oral antibiotic prescriptions are inappropriate [4]. Older patients may be disproportionately impacted by this practice. Outpatient antibiotic prescribing rates increased 30% among older adults from 2000 to 2010 [5]. The use of broad-spectrum antibiotics in older adults increased 68% during the same period [5].

Previous studies have examined the geographic variation of antibiotic prescribing in the United States in an effort to identify areas where antibiotic stewardship efforts are most needed. Utilizing data from 2011, Hicks et al. found that outpatient antibiotic use was highest in the South and lowest in the West [6]. At least two studies found a similar pattern in outpatient antibiotic prescribing among older adults. It is unknown if these patterns have continued in more recent years [4, 7]. Understanding the regional variations in antibiotic use among older patients is a necessary step in the development of targeted antimicrobial stewardship efforts.

The objective of this study was to describe regional variation in antibiotic prescribing and costs overall and by antibiotic class among Medicare Part D enrollees in 2013.

## Methods

This study utilized retrospective data from the 2013 Medicare Provider Utilization and Payment Data: Part D Prescriber Public Use File. The methodology for the development of this dataset has been described previously [7]. In brief, the dataset contains information on the approximately 35.7 million patients enrolled in the Medicare Part D prescription drug program. The data were collected using the Centers for Medicare and Medicaid Services (CMS) 2013 Medicare Part D Prescription Drug Event (PDE) Standard Analytic File, which includes PDEs received through June 30, 2014. This dataset contains information on 99.99% of all final-action claims submitted by Medicare Advantage Prescription Drug Plans and stand-alone Prescription Drug Plans. It includes the total number of prescriptions dispensed (including original prescriptions and refills) and total drug cost. Total drug cost represents the ingredient cost, dispensing fees, sales tax, and any applicable administration fees. The total number of enrolled beneficiaries was determined using a separate publicly available dataset, the Medicare Advantage/Part D Contract and Enrollment Data [8]. This dataset contains total enrollment numbers for both stand-alone Prescription Drug Plans (PDPs) and Medicare Advantage and Medicare Advantage Prescription Drug Plans (MAPDPs) at the state level.

We limited the dataset to all original and refill prescription claims for antibiotics. The PDE Standard Analytic File was searched manually by all authors for generic names corresponding to systemic antibiotics (excluding topical, otic, ophthalmic, or inhaled) or antibiotics used for the treatment of *Clostridium difficile* (i.e., oral vancomycin and fidaxomicin). The corresponding brand names for each identified generic antibiotic were then again searched manually to remove all non-systemic antibiotics. Any antibiotic that could not be definitively identified as systemic use only (e.g., tobramycin hydrochloride) was excluded from the final analysis. Additionally, antibiotics that have significant use for non-infectious indications (e.g., rifaximin) were also excluded. A listing of each included generic and brand name is included in Additional file 1. Antibiotics were categorized by therapeutic class, with parenteral antimicrobials considered separately from oral formulations. Data were analyzed descriptively by geographic region, as defined by the United States Census Bureau. The primary outcomes of interest were claims per enrollee and cost per enrollee. These outcomes were described overall and by antibiotic class. Population antibiotic claim rates and cost per enrollee were calculated by dividing the total number of PDEs or total cost, respectively, by the total number of enrollees in both PDPs and MAPDPs. The cost per claim was calculated by dividing the total cost for each antibiotic by the corresponding number of PDEs. We also calculated coefficients of variance, across both states and regions, for each of the primary outcomes. All analyses were performed using STATA v14.1 (StataCorp LP, College Station, TX). As all data were publicly available and non-identifiable, this study was deemed exempt by the institutional review board at The University of Texas MD Anderson Cancer Center. No external funding was obtained to complete this study.

## Results

**Fig. 1:**
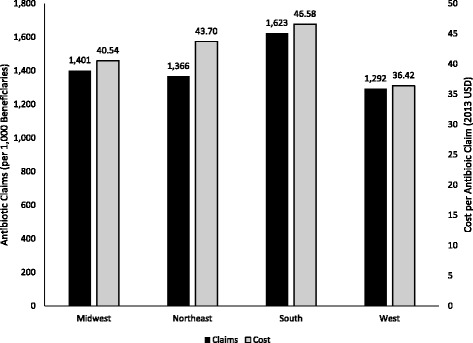
Antibiotic claims and cost for Medicare Part D Beneficiaries by U.S. census region (data from this study)

Over 54 million outpatient antibiotic claims were filed for Part D enrollees in 2013, representing 1,452 claims per 1,000 enrollees and more than $1.5 billion in total antibiotic expenditures. Across regions, antibiotic claims per 1,000 enrollees (Fig. 1) were highest in the South (1,623), followed by the Midwest (1,401), Northeast (1,366), and West (1,292). Average antibiotic costs per enrollee in each region (Fig. 1) were as follows: South ($46.58), Northeast ($43.70), Midwest ($40.54), and West ($36.42). The coefficients of variation across regions were 10% for claims per 1,000 enrollees and 10% for cost per enrollee.

**Fig. 2:**
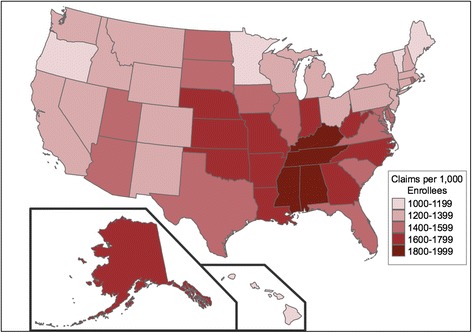
Antibiotic claims per 1,000 Medicare Part D enrollees, by state (data from this study, generated using http://choropleth.us/)

**Fig. 3:**
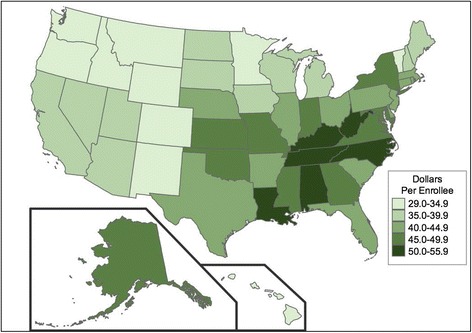
Antibiotic cost (in 2013 U.S. Dollars) per Medicare Part D enrollee, by state (data from this study, generated using http://choropleth.us/)

Across states, antibiotic claims per 1,000 enrollees (Fig. 3) were highest in Mississippi (1,911), Tennessee (1,866), Kentucky (1,837), and Alabama (1,836) and lowest in Minnesota (1,073), Hawaii (1,098), Oregon (1,120), and Vermont (1,147). Average antibiotic costs per enrollee (Fig. 2) were highest in Kentucky ($55.14), West Virginia ($54.43), and the District of Columbia ($53.82) and lowest in Hawaii ($29.41) and Vermont ($29.70). The coefficients of variation across states were 15% for claims per 1,000 enrollees and 16% for cost per enrollee; however, state-level variance in claims and costs tended to cluster non-randomly by geographic region (Fig. 2 and 3).

Table 1 describes the number of claims by antibiotic class. Fluoroquinolone antibiotics (12.2 million claims) were the most commonly prescribed class overall, accounting for 22% of all claims. Oral penicillins (10.0 million claims, 19%), macrolides (8.6 million claims, 16%), and oral cephalosporins (6.8 million claims, 13%) were also among the most commonly prescribed classes. This trend was consistent among all regions. Table 2 shows the most commonly prescribed antibiotic agents and the average cost per claim for each drug. Azithromycin was the most commonly prescribed antibiotic, with 7.2 million total claims and a rate of 194 claims per 1,000 enrollees. Doxycycline, with 3.2 million total claims at a total cost of $221 million, resulted in the highest cost per enrollee ($5.93). Fidaxomicin, with 7,857 claims and a total cost of $23 million, had the highest cost per claim ($2,948). Overall, the ten most frequently prescribed antibiotics accounted for 82% of all antibiotic prescription claims while the ten highest cost antibiotics represented 65% of all expenditures. A complete listing of claims and cost per antibiotic is available in Additional file 2.

**Table 1:** Claims per 1,000 enrollees by antibiotic class and region

	Region				
Class	Midwest	Northeast	South	West	Overall
Fluoroquinolone	298	303	379	287	326
Oral Penicillin	265	279	282	241	269
Macrolide	210	229	253	211	229
Oral Cephalosporin	188	158	201	169	183
SMX-TMP^a^	124	102	146	110	125
Tetracycline	101	85	115	83	99
Nitrofurantoin	63	61	79	58	67
Parenteral	30	33	35	28	32
Other	133	117	134	106	122

^a^SMX-TMP, sulfamethoxazole-trimethoprim

**Table 2:** Claims per 1,000 enrollees for most commonly prescribed antibiotics

Agent	Claims per 1,000 Enrollees	Cost per Claim (2013 USD^a^)
Azithromycin	194	12.33
Ciprofloxacin	185	7.28
Amoxicillin	154	5.61
Cephalexin	127	7.81
Sulfamethoxazole-Trimethoprim	125	6.73
Levofloxacin	112	14.77
Amoxicillin-Clavulanate	88	23.29
Doxycycline	86	68.92
Nitrofurantoin	67	45.67
Metronidazole	49	43.43

^a^2013 United States Dollars

## Discussion

This study described national antibiotic prescribing and costs among Medicare Part D enrollees in the United States by geographic region and found that the highest rate of prescribing occurs in the South census region. Importantly, this study represents one of the first to use publicly available data to characterize antibiotic prescribing trends at a national level, highlighting the potential for this dataset to serve as a new tool for researchers and public health authorities to understand broad trends in prescribing practices.

We found an overall antibiotic prescribing rate of 1,452 claims per 1,000 Part D enrollees, which is substantially higher than previous estimates of antibiotic use in this population. Among adults ≥65 years old, Hicks et al. reported 1,048 prescriptions per 1,000 persons in 2011 [6, 9]. In an analysis of National Ambulatory Medicare Care Survey (NAMCS) and National Hospital Ambulatory Medicare Care Survey (NHAMCS) data, Fleming-Dutra et al. estimated that there were just 617 antibiotic prescriptions per 1,000 persons ≥65 years old in 2010–2011 [4]. One reason for this difference is that these two studies analyzed data from the general population, while our study focused on Medicare Part D enrollees only. Furthermore, both studies relied on estimates derived from samples rather than direct measurements of claims, number of enrollees, and costs, potentially accounting for this variance.

Previous studies have characterized outpatient regional antibiotic prescribing trends in the United States and found similar prescribing patterns, with antibiotic use highest in the South region and lowest in the West [6, 7, 10]. Of particular relevance to our study, a subgroup analysis of patients ≥65 years old using 2011 data from the IMS Health Xponent database showed that prescription claims were highest in the south (1,160 prescriptions per 1,000 persons) and lowest in the West (882 prescriptions per 1,000).[6] The underlying causes of these geographic variations cannot be established from our data; however, at least one prior study found that regional patterns of antibiotic use were not significantly correlated with the prevalence of diagnoses for which antibiotics are commonly indicated (e.g., bacterial pneumonia) [7]. Differences in the occurrences of acute nasopharyngitis and nonspecific acute respiratory tract infections also failed to account for the variation [7]. We were unable to identify any regional epidemics which might influence the geographic distribution of antibiotic use. Additionally, such an epidemic would be an unlikely explanation for such variation given the similar findings of other studies conducted in previous years. We were also unable to correlate the level of antibiotic use to the proportion of Medicare enrollees in each region who qualified by age versus disability. A previous study found that black race and lack of health insurance were independent predictors of lower prescribing rates of broad-spectrum antibiotics for acute respiratory tract infections [11]. However, the proportion of Medicare enrollees who are black is highest in the South region, indicating that the high rate of antibiotic use occurs despite, rather than because of, differences in race [12]. A 2015 study found that areas with more prescribers per capita or a higher proportion of female, obese, or black patients had higher rates of antibiotic prescribing.[6] Conversely, areas with higher rates of 4-year college education and per capita income had lower rates of antibiotic prescribing [6]. At least one study has suggested that excessive antibiotic use is only one component of a more broad pattern low-quality prescribing in certain locations [13]. Other research suggests that variations in antibiotic use are likely due to a wide range of factors which vary between regions, including comorbidities, patient attitudes towards healthcare, formulary restrictions, physician values, practice volume, and medical culture in general [11]. Together, these findings suggest that any intervention aimed at unnecessary regional variations in antibiotic prescribing must address multiple societal and economic factors.

Appropriate antibiotic use is of paramount importance in elderly patients because of their higher risk for antibiotic-related adverse reactions (e.g., *Clostridium difficile* infection) and complications from drug-drug interactions, such as QT interval prolongation [14]. On the other hand, elderly patients are also more susceptible to adverse outcomes from untreated infections due to the presence of multiple comorbid conditions [15]. Consequently, it is critical to limit unnecessary antibiotic prescribing in this population while also ensuring that infections are properly treated with antibiotics when indicated.

The combination of drug use and cost data may allow for prioritization of any interventions aimed at reducing inappropriate antibiotic prescribing. The fluoroquinolones (i.e., levofloxacin, moxifloxacin, and ciprofloxacin) were among the most commonly prescribed agents in our study, with a prescribing rate of 326 claims per 1,000 enrollees, or 22% of all claims. This class of antibiotics is notable for its association with infrequent, yet severe, adverse effects which may be more likely to occur in elderly patients [16, 17]. Furthermore, they are implicated in the development of several types of antibiotic-resistant bacteria [18, 19]. The FDA recently advised that the risks of fluoroquinolones outweigh the benefits for patients with uncomplicated infections as many other treatment options exist [20]. Interventions aimed at limiting the use of fluoroquinolones in Medicare Part D enrollees may therefore be of particular benefit. Azithromycin, the most commonly prescribed single antibiotic at 194 claims per 1,000 enrollees, is associated with a small but significant increased risk of cardiac events compared to other antibiotics, with benefit outweighing risk when used appropriately [21, 22]. As recent studies have demonstrated that over half of all prescriptions for upper respiratory tract infections are inappropriate, interventions designed to decrease the use of azithromycin, which is commonly used for this purpose, may be similarly beneficial [7, 23]. Finally, doxycycline was the most costly antibiotic despite being only the eighth most commonly prescribed; as such, it may be a reasonable option for cost-containment interventions as the cost of long-available generic medications continues to increase [24].

Our findings indicate the need for stewardship interventions to promote appropriate antibiotic use where the burden is highest. Prescribing rates in regions with lower antibiotic use could serve as useful benchmarks when determining goals for reduction. Several types of interventions to reduce inappropriate antibiotic prescribing have been studied in the outpatient setting, including provider and patient education, provider feedback, communication skills training, restriction policies, and integration of rapid diagnostics [25]. All types appear effective, and none have been associated with adverse effects on patient outcomes or drug spending [25]. However, further study is needed on the feasibility and effectiveness of these interventions at the population level.

This study has several potential limitations. First, the dataset only contains patients enrolled under the Medicare Part D prescription drug program (i.e., about two-thirds of all Medicare beneficiaries), and may not represent antibiotic prescribing patterns for all Medicare beneficiaries or elderly patients. Furthermore, we are unable to determine the appropriateness of antibiotic prescribing using this dataset, and it is important to note that lower prescribing rates do not necessarily reflect more appropriate prescribing. Next, the data are limited to medications covered by Medicare Part D or those covered through supplemental benefits; therefore, the results might underestimate antibiotic prescribing in this population. Lastly, this study included a single year of data, which would not capture annual changes in prescribing patterns.

## Conclusions

In summary, antibiotic use among elderly Medicare Part D enrollees in the United States was highest in the South region, confirming the regional variation seen in prior years and in other populations. Fluoroquinolones were the most common antibiotics used in all regions. These patterns could be utilized in the development of systematic antimicrobial stewardship efforts.

## Additional files

Additional file 1: Generic and brand names of antibiotics included in the final analysis. (DOCX 15 kb)
Additional file 2: Number and cost of claims for included antibiotics. (DOCX 19 kb)
