# Supplementary material for: Regional variation in antibiotic prescribing among medicare part D enrollees, 2013
Source: BMC Infect Dis. 2016 Dec 9;16:744. doi: 10.1186/s12879-016-2091-0 (PMC5148872; doi:10.1186/s12879-016-2091-0)
Supplement: Additional file 1: — Generic and brand names of antibiotics included in the final analysis. (DOCX 15 kb) [file 12879_2016_2091_MOESM1_ESM.docx]

**Supplementary Appendix 1 – Generic and brand names of antibiotics included in the final analysis**

**Included generic names:**

| AMIKACIN SULFATE |
| --- |
| AMOXICILLIN |
| AMOXICILLIN-POTASSIUM CLAV |
| AMPICILLIN SODIUM |
| AMPICILLIN SODIUM-SULBACTAM NA |
| AMPICILLIN TRIHYDRATE |
| AZITHROMYCIN |
| AZTREONAM |
| AZTREONAM-DEXTROSE-WATER |
| BACITRACIN |
| CEFACLOR |
| CEFADROXIL HYDRATE |
| CEFAZOLIN SODIUM |
| CEFAZOLIN SODIUM-DEXTROSE-ISO |
| CEFDINIR |
| CEFDITOREN PIVOXIL |
| CEFEPIME HCL |
| CEFEPIME HCL-D5W |
| CEFEPIME HCL-DEXTROSE- ISO-OSM |
| CEFIXIME |
| CEFOTAXIME SODIUM |
| CEFOTETAN DISODIUM |
| CEFOXITIN SODIUM |
| CEFOXITIN SODIUM-DEXTROSE-ISO |
| CEFPODOXIME PROXETIL |
| CEFPROZIL |
| CEFTAROLINE FOSAMIL ACETATE |
| CEFTAZIDIME NA-DEXTROSE-ISO |
| CEFTAZIDIME PENTAHYDRATE |
| CEFTRIAXONE NA-DEXTROSE-ISO |
| CEFTRIAXONE SODIUM |
| CEFUROXIME AXETIL |
| CEPHALEXIN |
| CIPROFLOXACIN |
| CIPROFLOXACIN HCL |
| CIPROFLOXACIN LACTATE |
| CIPROFLOXACIN LACTATE-D5W |
| CIPROFLOXACIN-CIPROFLOXA HCL |
| CLARITHROMYCIN |
| CLINDAMYCIN HCL |
| CLINDAMYCIN PALMITATE HCL |
| CLINDAMYCIN PHOSPHATE |
| CLINDAMYCIN PHOSPHATE-D5W |
| COLISTIN (COLISTIMETHATE NA) |
| DAPTOMYCIN |
| DICLOXACILLIN SODIUM |
| DORIPENEM |
| DOXYCYCLINE CALCIUM |
| DOXYCYCLINE HYCLATE |
| DOXYCYCLINE MONOHYDRATE |
| ERTAPENEM SODIUM |
| ERYTHROMYCIN BASE |
| ERYTHROMYCIN ETHYLSUCCINATE |
| ERYTHROMYCIN LACTOBIONATE |
| ERYTHROMYCIN STEARATE |
| ETHAMBUTOL HCL |
| FIDAXOMICIN |
| FOSFOMYCIN TROMETHAMINE |
| GEMIFLOXACIN MESYLATE |
| IMIPENEM-CILASTATIN SODIUM |
| ISONIAZID |
| LEVOFLOXACIN |
| LEVOFLOXACIN-D5W |
| LINCOMYCIN HCL |
| LINEZOLID |
| MEROPENEM |
| METRONIDAZOLE |
| METRONIDAZOLE-SODIUM CHLORIDE |
| MINOCYCLINE HCL |
| MINOCYCLINE HCL MICROSPHERES |
| MOXIFLOXACIN HCL |
| MOXIFLOXACIN IN NACL (ISO-OSM) |
| NAFCILLIN IN DEXTROSE-ISO-OSM |
| NAFCILLIN SODIUM |
| NITROFURANTOIN |
| NITROFURANTOIN MACROCRYSTAL |
| NITROFURANTOIN MONOHYD-M-CRYST |
| NORFLOXACIN |
| OFLOXACIN |
| OXACILLIN SODIUM |
| OXACILLIN SODIUM-DEXTROSE-ISO |
| PEN G BENZ-PEN G PROCAINE |
| PEN G POT-DEXTROSE-WATER |
| PENICILLIN G BENZATHINE |
| PENICILLIN G POTASSIUM |
| PENICILLIN G SODIUM |
| PENICILLIN V POTASSIUM |
| PIPERACILLIN SODIUM-TAZOBACTAM |
| PIPERACILLIN-TAZO-DEXTROSE-ISO |
| POLYMYXIN B SULFATE |
| RIFABUTIN |
| RIFAMPIN |
| RIFAPENTINE |
| STREPTOMYCIN SULFATE |
| SULFAMETHOXAZOLE-TRIMETHOPRIM |
| TELITHROMYCIN |
| TETRACYCLINE HCL |
| TICARCILLIN-K CLAVULANATE |
| TIGECYCLINE |
| TRIMETHOPRIM |
| VANCOMYCIN HCL |
| VANCOMYCIN HCL-D5W |
| VANCOMYCIN IN DEXTROSE-ISO-OSM |

**Included brand names**

| AMIKACIN SULFATE |
| --- |
| AMOX TR-POTASSIUM CLAVULANATE |
| AMOXICILLIN |
| AMOXICILLIN-CLAVULANATE ER |
| AMPICILLIN SODIUM |
| AMPICILLIN TRIHYDRATE |
| AMPICILLIN-SULBACTAM |
| AUGMENTIN |
| AUGMENTIN XR |
| AVELOX |
| AVELOX ABC PACK |
| AVELOX IV |
| AZACTAM |
| AZACTAM-ISO-OSMOTIC DEXTROSE |
| AZITHROMYCIN |
| AZTREONAM |
| BACIIM |
| BACITRACIN |
| BACTRIM |
| BACTRIM DS |
| BIAXIN |
| BIAXIN XL |
| BICILLIN C-R |
| BICILLIN L-A |
| CEFACLOR |
| CEFACLOR ER |
| CEFADROXIL |
| CEFAZOLIN |
| CEFAZOLIN SODIUM |
| CEFDINIR |
| CEFDITOREN PIVOXIL |
| CEFEPIME |
| CEFEPIME HCL |
| CEFEPIME-DEXTROSE |
| CEFOTAXIME SODIUM |
| CEFOTETAN |
| CEFOXITIN |
| CEFOXITIN SODIUM |
| CEFPODOXIME PROXETIL |
| CEFPROZIL |
| CEFTAZIDIME |
| CEFTIN |
| CEFTRIAXONE |
| CEFUROXIME |
| CEPHALEXIN |
| CIPRO |
| CIPROFLOXACIN |
| CIPROFLOXACIN ER |
| CIPROFLOXACIN HCL |
| CIPROFLOXACIN-D5W |
| CLAFORAN |
| CLARITHROMYCIN |
| CLARITHROMYCIN ER |
| CLEOCIN |
| CLEOCIN HCL |
| CLEOCIN PALMITATE |
| CLEOCIN PHOSPHATE |
| CLEOCIN PHOSPHATE IN D5W |
| CLINDACIN P |
| CLINDAMYCIN HCL |
| CLINDAMYCIN PALMITATE HCL |
| CLINDAMYCIN PEDIATRIC |
| CLINDAMYCIN PHOSPHATE |
| CLINDAMYCIN PHOSPHATE-D5W |
| COLISTIMETHATE |
| COLISTIMETHATE SODIUM |
| COLY-MYCIN M PARENTERAL |
| CUBICIN |
| DICLOXACILLIN SODIUM |
| DIFICID |
| DORIBAX |
| DOXY 100 |
| DOXYCYCLINE HYCLATE |
| DOXYCYCLINE MONOHYDRATE |
| E.E.S. 200 |
| E.E.S. 400 |
| ERY-TAB |
| ERYPED 200 |
| ERYPED 400 |
| ERYTHROCIN LACTOBIONATE |
| ERYTHROCIN STEARATE |
| ERYTHROMYCIN |
| ERYTHROMYCIN ETHYLSUCCINATE |
| ETHAMBUTOL HCL |
| FACTIVE |
| FLAGYL |
| FLAGYL ER |
| FORTAZ |
| FORTAZ IN ISO-OSMOTIC DEXTROSE |
| FURADANTIN |
| ILOTYCIN |
| IMIPENEM-CILASTATIN SODIUM |
| INVANZ |
| ISONIAZID |
| KEFLEX |
| KETEK |
| LEVAQUIN |
| LEVOFLOXACIN |
| LEVOFLOXACIN-D5W |
| LINCOCIN |
| MACROBID |
| MACRODANTIN |
| MAXIPIME |
| MEROPENEM |
| MERREM |
| METRO IV |
| METRONIDAZOLE |
| MINOCIN |
| MINOCYCLINE HCL |
| MONODOX |
| MONUROL |
| MOXATAG |
| MYAMBUTOL |
| MYCOBUTIN |
| NAFCILLIN |
| NAFCILLIN SODIUM |
| NITROFURANTOIN |
| NITROFURANTOIN MONO-MACRO |
| NOROXIN |
| OFLOXACIN |
| OXACILLIN |
| OXACILLIN SODIUM |
| PCE |
| PENICILLIN G POTASSIUM |
| PENICILLIN G SODIUM |
| PENICILLIN GK-ISO-OSM DEXTROSE |
| PENICILLIN V POTASSIUM |
| PFIZERPEN |
| PIPERACILLIN-TAZOBACTAM |
| POLYMYXIN B SULFATE |
| PRIFTIN |
| PRIMAXIN |
| PRIMSOL |
| RIFADIN |
| RIFAMPIN |
| ROCEPHIN |
| SPECTRACEF |
| STREPTOMYCIN SULFATE |
| SULFAMETHOXAZOLE-TRIMETHOPRIM |
| SUPRAX |
| TAZICEF |
| TEFLARO |
| TETRACYCLINE HCL |
| TIMENTIN |
| TRIMETHOPRIM |
| TYGACIL |
| UNASYN |
| VANCOCIN HCL |
| VANCOMYCIN |
| VANCOMYCIN HCL |
| VANCOMYCIN-D5W |
| VIBRAMYCIN |
| ZITHROMAX |
| ZITHROMAX TRI-PAK |
| ZMAX |
| ZOSYN |
| ZYVOX |
