# Supplementary material for: Regional variation in antibiotic prescribing among medicare part D enrollees, 2013
Source: BMC Infect Dis. 2016 Dec 9;16:744. doi: 10.1186/s12879-016-2091-0 (PMC5148872; doi:10.1186/s12879-016-2091-0)
Supplement: Additional file 2: — Number and cost of claims for included antibiotics. (DOCX 19 kb) [file 12879_2016_2091_MOESM2_ESM.docx]

**Supplementary Appendix 2 – Number and cost of claims for included antibiotics**

| Generic Name | Total Number of Drug Claims | Total Drug Expenditure (2013 USD) | Antibiotic Claims / 1,000 Part D Beneficiaries | Average Cost / Claim (2013 USD) |
| --- | --- | --- | --- | --- |
| AMIKACIN SULFATE | 14,023 | $963,828.78 | 0.38 | $68.73 |
| AMOXICILLIN | 5,768,071 | $32,346,798.06 | 154.34 | $5.61 |
| AMOXICILLIN-POTASSIUM CLAV | 3,301,675 | $76,897,214.16 | 88.35 | $23.29 |
| AMPICILLIN SODIUM | 7,774 | $1,544,275.61 | 0.21 | $198.65 |
| AMPICILLIN SODIUM-SULBACTAM NA | 10,350 | $1,451,465.99 | 0.28 | $140.24 |
| AMPICILLIN TRIHYDRATE | 169,481 | $1,231,113.47 | 4.53 | $7.26 |
| AZITHROMYCIN | 7,236,446 | $89,221,375.46 | 193.63 | $12.33 |
| AZTREONAM | 7,213 | $3,785,366.37 | 0.19 | $524.80 |
| BACITRACIN | 124,430 | $7,544,869.35 | 3.33 | $60.64 |
| CEFACLOR | 23,551 | $1,289,262.13 | 0.63 | $54.74 |
| CEFADROXIL HYDRATE | 166,783 | $3,249,477.62 | 4.46 | $19.48 |
| CEFAZOLIN | 25,106 | $1,631,766.15 | 0.67 | $65.00 |
| CEFDINIR | 692,311 | $28,706,442.42 | 18.52 | $41.46 |
| CEFDITOREN PIVOXIL | 427 | $114,854.91 | 0.01 | $268.98 |
| CEFEPIME | 51,697 | $9,734,196.15 | 1.38 | $188.29 |
| CEFIXIME | 25,459 | $4,783,822.26 | 0.68 | $187.90 |
| CEFOTAXIME SODIUM | 394 | $26,168.52 | 0.01 | $66.42 |
| CEFOTETAN DISODIUM | 339 | $48,090.21 | 0.01 | $141.86 |
| CEFOXITIN | 2,264 | $546,339.08 | 0.06 | $241.32 |
| CEFPODOXIME PROXETIL | 114,505 | $11,713,625.25 | 3.06 | $102.30 |
| CEFPROZIL | 67,844 | $4,177,227.94 | 1.82 | $61.57 |
| CEFTAROLINE FOSAMIL ACETATE | 6,647 | $3,538,382.47 | 0.18 | $532.33 |
| CEFTAZIDIME | 17,350 | $2,271,018.44 | 0.46 | $130.89 |
| CEFTRIAXONE | 290,663 | $23,202,403.25 | 7.78 | $79.83 |
| CEFUROXIME AXETIL | 1,000,384 | $16,255,020.47 | 26.77 | $16.25 |
| CEPHALEXIN | 4,735,153 | $36,971,273.10 | 126.70 | $7.81 |
| CIPROFLOXACIN | 6,928,781 | $50,444,488.34 | 185.40 | $7.28 |
| CIPROFLOXACIN LACTATE | 5,162 | $333,564.87 | 0.14 | $64.62 |
| CLARITHROMYCIN | 485,615 | $37,216,685.11 | 12.99 | $76.64 |
| CLINDAMYCIN | 1,830,305 | $24,347,593.20 | 48.97 | $13.30 |
| CLINDAMYCIN PHOSPHATE | 323,476 | $21,800,570.72 | 8.66 | $67.39 |
| COLISTIN (COLISTIMETHATE NA) | 3,950 | $2,755,415.90 | 0.11 | $697.57 |
| DAPTOMYCIN | 39,915 | $75,320,111.41 | 1.07 | $1,887.01 |
| DICLOXACILLIN | 59,849 | $1,409,211.18 | 1.60 | $23.55 |
| DORIPENEM | 3,250 | $1,681,831.68 | 0.09 | $517.49 |
| DOXYCYCLINE | 3,205,130 | $220,912,849.95 | 85.76 | $68.92 |
| ERTAPENEM SODIUM | 68,422 | $28,954,862.11 | 1.83 | $423.18 |
| ERYTHROMYCIN | 853,709 | $33,835,608.03 | 22.84 | $39.63 |
| ETHAMBUTOL HCL | 47,878 | $4,482,737.53 | 1.28 | $93.63 |
| FIDAXOMICIN | 7,857 | $23,162,722.07 | 0.21 | $2,948.04 |
| FOSFOMYCIN TROMETHAMINE | 16,944 | $2,353,297.28 | 0.45 | $138.89 |
| GEMIFLOXACIN MESYLATE | 1,124 | $386,363.11 | 0.03 | $343.74 |
| IMIPENEM-CILASTATIN SODIUM | 20,505 | $4,482,881.19 | 0.55 | $218.62 |
| ISONIAZID | 52,084 | $619,476.64 | 1.39 | $11.89 |
| LEVOFLOXACIN | 4,168,763 | $61,567,371.43 | 111.55 | $14.77 |
| LEVOFLOXACIN-D5W | 16,191 | $914,528.58 | 0.43 | $56.48 |
| LINCOMYCIN HCL | 221 | $11,494.46 | 0.01 | $52.01 |
| LINEZOLID | 54,408 | $122,094,154.16 | 1.46 | $2,244.05 |
| MEROPENEM | 38,977 | $11,314,113.42 | 1.04 | $290.28 |
| METRONIDAZOLE | 1,837,225 | $79,789,300.40 | 49.16 | $43.43 |
| METRONIDAZOLE-SODIUM CHLORIDE | 4,827 | $105,244.45 | 0.13 | $21.80 |
| MINOCYCLINE HCL | 495,708 | $12,660,150.63 | 13.26 | $25.54 |
| MOXIFLOXACIN HCL | 330,933 | $61,781,496.59 | 8.86 | $186.69 |
| MOXIFLOXACIN IN NACL (ISO-OSM) | 1,205 | $218,682.43 | 0.03 | $181.48 |
| NAFCILLIN | 6,781 | $5,371,786.76 | 0.18 | $792.18 |
| NITROFURANTOIN | 2,511,183 | $114,678,655.29 | 67.19 | $45.67 |
| NORFLOXACIN | 4,276 | $527,568.11 | 0.11 | $123.38 |
| OFLOXACIN | 742,757 | $8,568,428.57 | 19.87 | $11.54 |
| OXACILLIN | 3,020 | $2,455,166.06 | 0.08 | $812.97 |
| PEN G BENZ-PEN G PROCAINE | 176 | $38,150.33 | 0.00 | $216.76 |
| PEN G POT-DEXTROSE-WATER | 359 | $151,084.58 | 0.01 | $420.85 |
| PENICILLIN G BENZATHINE | 4,092 | $1,171,679.00 | 0.11 | $286.33 |
| PENICILLIN G POTASSIUM | 3,123 | $694,143.14 | 0.08 | $222.27 |
| PENICILLIN G SODIUM | 154 | $162,012.34 | 0.00 | $1,052.03 |
| PENICILLIN V POTASSIUM | 744,702 | $7,080,822.35 | 19.93 | $9.51 |
| PIPERACILLIN | 51,868 | $13,078,440.39 | 1.39 | $252.15 |
| POLYMYXIN B SULFATE | 5,175 | $378,998.37 | 0.14 | $73.24 |
| RIFABUTIN | 6,747 | $4,725,613.55 | 0.18 | $700.40 |
| RIFAMPIN | 94,098 | $6,394,284.58 | 2.52 | $67.95 |
| RIFAPENTINE | 520 | $68,720.52 | 0.01 | $132.15 |
| STREPTOMYCIN SULFATE | 226 | $24,758.99 | 0.01 | $109.55 |
| SULFAMETHOXAZOLE-TRIMETHOPRIM | 4,665,456 | $31,407,156.03 | 124.84 | $6.73 |
| TELITHROMYCIN | 104 | $63,389.76 | 0.00 | $609.52 |
| TETRACYCLINE HCL | 3,643 | $190,131.86 | 0.10 | $52.19 |
| TICARCILLIN-K CLAVULANATE | 27 | $8,259.80 | 0.00 | $305.92 |
| TIGECYCLINE | 11,004 | $9,154,476.52 | 0.29 | $831.92 |
| TRIMETHOPRIM | 273,891 | $4,546,908.10 | 7.33 | $16.60 |
| VANCOMYCIN HCL | 347,354 | $124,588,570.66 | 9.29 | $358.68 |
| VANCOMYCIN IV | 20,061 | $2,545,530.19 | 0.54 | $126.89 |
